# Using a causal decomposition approach to estimate the contribution of employment to differences in mental health profiles between men and women

**DOI:** 10.1016/j.ssmph.2024.101718

**Published:** 2024-10-12

**Authors:** Christa Orchard, Elizabeth Lin, Laura Rosella, Peter M. Smith

**Affiliations:** a Dalla Lana School of Public Health, University of Toronto, Toronto, Ontario, Canada; b Institute for Work & Health, Toronto, Ontario, Canada; c ICES, Ontario, Canada; d Centre for Addiction and Mental Health, Toronto, Ontario, Canada; e Department of Psychiatry, University of Toronto, Toronto, Ontario, Canada; f Institute for Better Health, Trillium Health Partners, Mississauga, Ontario, Canada; g Temerty Faculty of Medicine, University of Toronto, Toronto, Ontario, Canada

**Keywords:** Mental health, Employment, Well-being, Causal analysis, Social determinants of health, Gender role

## Abstract

**Background:**

Mental health disorders are known to manifest differently in men and women, however our understanding of how gender interacts with mental health and well-being as a broader construct remains limited. Employment is a key determinant of mental health and there are historical differences in occupational roles among men and women that continue to influence working lives (Bonde, 2008; Cabezas-Rodríguez, Utzet, & Bacigalupe, 2021; Drolet, 2022; Gedikli, Miraglia, Connolly, Bryan, & Watson, 2023; Moyser, 2017; Niedhammer, Bertrais, & Witt, 2021; Stier & Yaish, 2014; Van der Doef & Maes, 1999). This study aims to explore differences in multidimensional mental health between men and women, and to quantify how these differences may change if women had the same employment characteristics as men.

**Methods:**

Working-age adults (25–64) were identified through a household survey in Ontario, Canada during 2012. We created multifaceted measures of employment to capture both employment and job quality, as well as multidimensional mental health profiles that capture mental health disorders and well-being using survey data. A causal decomposition approach with Monte Carlo simulation methods estimated the change in differences in mental health profiles between men and women, if women had the same employment characteristics as men.

**Results:**

Among 2458 eligible respondents, women were more likely to exhibit clinical mood disorders compared to men, with men more likely to exhibit absence of flourishing without a diagnosable disorder. Among those who were flourishing, women more often expressed at least some life stress compared to men. When women were assigned men's employment characteristics, which amounted to an increase in employment and higher quality employment, some of the gender differences in risk of clinical mood disorder decreased. However, differences between men and women in the remaining mental health profiles increased.

**Conclusions:**

This study provided an estimate of the contribution of employment to the observed differences in multidimensional mental health between men and women. This adds to the literature by including a broader range of mental health indicators than disorders alone, and by formalizing the causal framework used to study these relationships.

## Highlights

• Gender differences in mental health are complex and multidimensional.
• Causal decomposition can estimate the contribution of employment to these differences.
• Men and women exhibited different combinations of flourishing, life stress and clinical disorders.
• Gender differences in clinical mental health disorders were partly explained by employment.
• Gender differences in positive mental health and well-being were partly suppressed by gender differences in employment.

## Abbreviations:

CCHS-MH: Canadian Community Health Survey, mental health edition
PAM: Partitioning Around Medoids
RD: Risk Difference

## 1 Introduction

Differences in mental health and wellbeing between men and women are complex and multifaceted. Clinically diagnosable mental health disorders and symptoms often manifest differently in men and women. Women are more susceptible to affective disorders including mood and anxiety disorders, and men more often exhibit externalizing disorders such as substance use disorders and antisocial personality disorders (Kessler & Zhao, 1999; Seedat et al., 2009). However, mental health is a multidimensional construct that goes beyond simply the absence of mental illness. Multiple other facets of mental health have been linked to substantial impairment and help-seeking including life stress, self-rated mental health, and psychological distress (Fleury, Ngui, Bamvita, Grenier, & Caron, 2014; Slavich, 2016). In addition, a greater focus has been placed in recent years on positive mental health and ability to achieve ‘flourishing’ as an important population mental health outcome in and of itself, which has also been shown to have protective effects on all-cause mortality, suicide and mental illness (Grant et al., 2013, Guille, & Sen, 2013; Corey L. M. Keyes et al., 2012; Corey L. M Keyes & Simoes, 2012). Our understanding of the differences between women and men in mental health and well-being as a broader construct remains limited.

Some evidence suggests men are more likely to be flourishing in their mental health compared to women (Kessler & Zhao, 1999; Seedat et al., 2009). However, a Canadian study reported no differences between men and women in ‘complete mental health’, defined as being flourishing and absent of mental illness (Gilmour, 2014). Similarly some research has shown that women are more likely to express life satisfaction compared to men, whereas others have identified minimal gender differences (Batz-Barbarich, Tay, Kuykendall, & Cheung, 2018; OECD, 2013). These different dimensions of mental health also interact with each other and may do so differently among men and women. For example, the presence or absence of flourishing among those with mental illness is an important predictor of long-term outcomes including resilience and suicidal behaviour (Corey L. M. Keyes et al., 2012; Provencher & Keyes, 2011). Our current understanding of differences in multidimensional mental health between men and women is limited given that most research in this area focus only on single indicators.

Further, while understanding gender disparities in mental health is important, we cannot perform a population-level intervention on social identity characteristics, such as gender, for the purpose of preventing health outcomes (VanderWeele & Robinson, 2014). Unpacking the factors that underpin the relationship between gender and mental health has higher relevance to policy and interventions that may aim to reduce these disparities. Any differences between men and women in mental health and wellbeing are likely underpinned by a range of factors, both biological (sex) and social (gender). Gender includes different dimensions such as identity, roles, and expectations. It is also important to note that gender holds diversity that goes beyond the binary categories of man and woman, however this work attempts to build on existing research that compared women relative to men.

Historically, men and women have held different societal roles and related expectations. An important part of this is employment and working lives. The division of labour within families has traditionally been distributed differently among men and women; with women less likely to participate in the labour market, and more likely to take on household responsibilities and primary caregiving roles (Moyser, 2017). While, over time, this has changed with more women entering and maintaining employment, a gender gap in employment remains (Drolet, 2022; Moyser, 2017). Within the labour market there are also differences in quality of employment between men and women, with men more often inhabiting roles with greater opportunities for training and promotion, and higher job complexity and autonomy compared to women (Cabezas-Rodríguez et al., 2021; Stier & Yaish, 2014).

Work is a key social determinant of health. Unemployment, precarious work, psychosocial work exposures, job quality and occupational work hazards are some of the aspects of working lives that have been identified as drivers of health outcomes, captured most acutely through their impacts on all cause mortality (Amick et al., 2002; Frank et al., 2023). Working conditions are largely influenced by socioeconomic conditions, which have created widely documented inequities across groups such as gender, ethnicity, age and migration status (Frank et al., 2023). Differences in working lives are therefore key to understanding social inequities in health.

As a result, understanding the contribution of working lives to gender disparities in mental health is of high interest and importance. Unemployment is an established risk factor for mental health problems including common mental health disorders, life stress and lower life satisfaction (Cabezas-Rodríguez et al., 2021; Gedikli et al., 2023). While low job control has been linked to poorer mental health outcomes including stress and depression (Bonde, 2008; Niedhammer et al., 2021; Van der Doef & Maes, 1999). Given the established relationships between gender, work and mental health, it is possible that differences in working lives between men and women could explain some of the differences we see in mental health between men and women.

An increasing number of studies have attempted to estimate the contribution of employment to gender differences in mental health (Amroussia, Gustafsson, & Mosquera, 2017; Hwang & Shin, 2023; Milner et al., 2020; Peckham, Seixas, de Castro, & Hajat, 2022; Platt, Bates, Jager, McLaughlin, & Keyes, 2020). These studies generally observe that gender differences in employment explain some of the increased prevalence of mental health disorders among women relative to men. However, these studies are limited in two important ways. Firstly, few take a causal approach, which is important given that without proper consideration of confounding covariates we cannot assess to what extent the explanatory contribution is due to other external factors (Jackson & VanderWeele, 2018). Studies that have attempted to take a causal approach have used mediation methods that do not adequately account for exposure-induced mediator-outcome confounders that are typical of social exposures such as gender, given it's broad-ranging impact on social and health outcomes (Jackson & VanderWeele, 2018). Second, studies in this area have typically focused on either employment (versus unemployment) or job quality among the employed rather than examining both, which we refer to here on in as ‘employment profile’. Similarly, these studies typically focus on single indicators of mental health such as depression rather than incorporating mental health as a multidimensional construct.

Using a causal decomposition approach, this study aims firstly to describe differences between men and women across multidimensional mental health profiles. Secondly, we aim to quantify the differences between men and women in mental health profiles if women had the employment profile distribution of men.

## 2 Materials and methods

### 2.1 Data sources and population

The primary data source for this study is the 2012 Canadian Community Health Survey, mental health edition (CCHS-MH), a cross-sectional survey disseminated to a representative sample of Canadians living in private dwellings aged 15 years and over (Statistics Canada, 2012). All residents of Ontario aged 25–64 who completed the 2012 CCHS-MH survey were included. Those aged under 25 or over 64 were excluded to focus on a typical ‘working age’ population.

### 2.2 Measurement

#### 2.2.1 Group- gender

Measured in the CCHS-MH, using a binary indicator coded by the interviewer. While described by the CCHS as biological sex, given it is interviewer-assessed, not necessarily with confirmation from the respondent, it is more likely to represent gender presentation. Gender differences using this definition are likely to reflect a mixture of social (gender) and biological (sex) differences.

#### 2.2.2 Explanatory variable – employment profile

We created a variable indicating both employment status and quality of employment with 7 levels. In the CCHS-MH all respondents were asked about their paid work activities in the prior 2 weeks. Participants who did not report any work during this time nor absence from a job due to sickness or leave were assumed unemployed. This group were also asked about their job search activities in the prior 2 weeks. To distinguish between those who were unemployed by choice and/or likely being financially supported through means other than personal income, we divided the unemployed group into 1) Unemployed and looking for work, 2) Unemployed and unable to work, 3) Unemployed and not looking for work. Among those who did report paid employment in the 2 weeks prior to the survey, we used 5 measures of job control, including whether their primary job requires them to learn new things, requires a high level of skill, allows freedom in how they do their job, requires repetitive tasks, and whether they have input into their own job (Brisson & Larocque, 2001). Each answer was assigned a value of 1–5 from strongly agree to strongly disagree (reverse coded for repetitive tasks), the scores were summed and divided into quartiles to create 4 groups 4) Quartile 1 (highest job control), 5) Quartile 2, 6) Quartile 3, and 7) Quartile 4 (lowest job control). All CCHS-MH questions used to create this variable are available in appendix B.

#### 2.2.3 Outcome- mental health profile

**Fig. 1:**
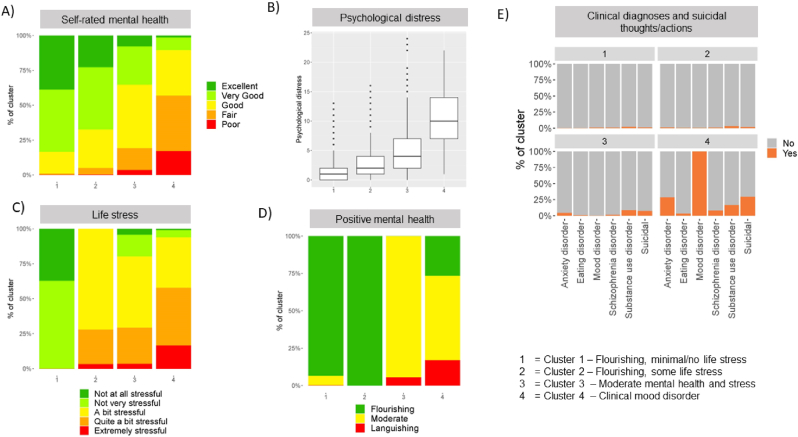
– Mental health profiles by input variables.

To summarise mental health as a multidimensional concept, we used a data-driven approach whereby common combinations of different mental health dimensions were identified. These ‘mental health profiles’ were generated as part of previous work using a Partitioning Around Medoids (PAM) clustering algorithm with Gower's proximity function based on the following input variables from the CCHS-MH: mood disorder, anxiety disorder, substance use disorder, schizophrenia or psychosis, eating disorder, suicidal thoughts or attempts, self-rated mental health, life stress and positive mental health. Details on the clustering procedure, measures used, and operationalization of these variables are described in prior work (Orchard, Lin, Rosella, & Smith, 2024), some details are also available in appendix C. The four mental health profiles identified, displayed by their five components in Fig. 1, include 1) Flourishing with minimal/no life stress, 2) Flourishing with some life stress, 3) Moderate mental health and stress, and 4) Clinical mood disorder.

**Fig. 2:**
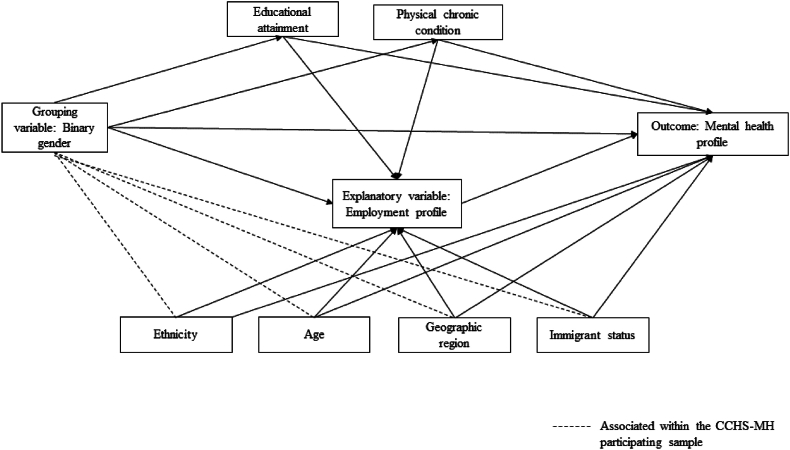
– Directed acyclic graph.

*Confounders:* A Directed Acyclic Graph outlining the expected relationships among the study variables is available in Fig. 2. It is expected that certain sociodemographic groups are more likely to participate in the CCHS-MH sample, and the factors predicting participation could be different among men and women, therefore inducing a relationship between sociodemographic characteristics (including age, ethnicity and geographic region) and gender within this sample. There are also a range of factors that are linked to mental health and employment including the aforementioned sociodemographic characteristics, as well as physical health (measured as presence or absence of any physical chronic condition), highest level of educational attainment, and having immigrated to Canada in the past 10 years.

#### 2.2.4 Analyses

To understand the contribution of employment characteristics to gender differences in mental health profiles, we used a causal decomposition approach (Jackson & VanderWeele, 2018). In this context, we are in effect simulating a hypothetical intervention whereby women are assigned randomly selected employment profile values from the distribution of employment profiles among men with similar confounder values (counterfactual distribution). By doing so we can examine differences in mental health profiles between men and women, if we were to be able to shift the distribution of employment characteristics among women to be similar to that of men, within groups of the confounding covariates. Through taking a causal approach to this work we have also assessed the requirements for causality including conditional exchangeability, positivity and consistency. By using a simulation approach, as opposed to a mediation approach whereby natural direct and indirect pathways are decomposed, we can also include exposure induced mediator to outcome confounders, of which we have two in Figure One (education, and physical chronic conditions). A more detailed discussion of the causal assumptions in this work is available in appendix A.

We implemented the causal decomposition using Monte Carlo integration and the parametric g-formula, using a process first outlined by Sudharsanan & Bijlsma (Sudharsanan & Bijlsma, 2021). In implementing this process, first a model for the explanatory variable (employment characteristics) was run using multinomial logistic regression, including gender and all confounders. The same process was repeated for the outcome, this time including gender, all confounders plus employment profile and an interaction term between employment and gender. These two models were used to created predicted probabilities for each of the simulated populations described below.

A Monte Carlo simulation loop was run to estimate the “natural course”, in other words the simulated outcome for each individual given their actual gender and covariate values. During each Monte Carlo simulation loop, an employment profile was drawn for each individual, with probability of selection based on their predicted probabilities of each employment profile given their actual sex and confounder values (using the logistic regression model calculated previously). The same process was completed to draw a mental health profile for each individual. The risk difference was then computed as the difference in the prevalence of each mental health profile between women and men, when their simulated explanatory variable and outcome followed a natural course.

We then selected all women in the sample and assigned them to have the counterfactual gender ‘man’. We repeated the simulation procedure whereby we drew an employment profile for the women informed by predicted probabilities given their actual confounder values, but this time defining their gender as men. We then reassigned gender back to women for this group and simulated their outcome based on predicted probabilities given their actual gender and confounders, plus their counterfactual simulated explanatory variable (employment profile). We then computed the counterfactual risk difference as the difference in the prevalence of each mental health profile among women compared to men, where both women and men have been assigned men's distribution of the explanatory variable.

This Monte Carlo simulation process was repeated 20 times and the risk difference was averaged across simulations. To account for missing data due to item-level non-response, we used a multiple imputation approach, whereby 20 imputed datasets were created with different missing values generated using the other covariates, the analyses described above was repeated using each of these 20 datasets and the averaged risk differences from the Monte Carlo process were also averaged across the imputed samples. The entire process was then repeated 100 times with different bootstrapped samples, the imputation procedure was performed within each bootstrap using the method described as ‘Boot MI’ by Schomaker & Heumann (Schomaker & Heumann, 2018). Finally, the average risk difference for the natural course and counterfactual scenarios across the bootstrap samples was computed for each level of the outcome (for each mental health profile), along with the 2.5th and 97.5th percentile of bootstrap estimates as confidence limits. The percentage change was calculated as the difference in effect estimates when using counterfactual employment profiles compared to natural employment profiles. There are no specific recommendations for the number of repetitions of each procedure. Due to using Monte Carlo repetitions within imputed datasets within bootstrapped samples, the computational processing powered required for this analysis was high, we therefore selected the maximum number of repetitions of each procedure we were able to run given computing resources. All analyses were completed in R version 4.1.2 (code available upon request).

## 3 Results

Of the 4159 Ontario residents who completed the CCHS-MH, 1701 (40.1%) were excluded due to being under 25 or over 65 the time of interview. This left 2458 eligible individuals. Of the 2458 eligible respondents, 328 (13.3%) were missing data on one or more measures. The variable with the largest degree of missing data was mental health profile (6.8%) followed by highest level of education (4.6%). As described in the methods section, missing data was accounted for using multiple imputation.

Table 1 displays the sociodemographic characteristics of the analytical sample by gender. Overall, the average age of the sample was 46, with representation across age groups, the majority (83.2%) resided in urban areas, most had high levels of education with almost three quarters reporting postsecondary graduation and nearly four in five self-identified as white. There were some gender differences, women were on average slightly younger than men, with more younger adults under 35 (23.6% vs 19.6%). Women were also more likely to be unemployed (29.3% vs 20.1%), slightly more likely to be lower earners of less than $40,000 per year (28.9% vs 22.8%), slightly more likely to be a recent immigrant (7.1% vs 5.7%), and slightly more likely to report a physical chronic condition (56.5% vs 51.0%).

In Table 2, employment profiles and mental health profiles are displayed for men and women. Men were not just more often employed overall but also more likely to be in the highest quartiles of job control compared to women (41.6% vs 33.6%). While women were more likely to be unemployed overall, a similar proportion of men and women reported job seeking activities and inability to work, whereas a higher proportion of women were unemployed and not looking for work compared to men (19.5% vs 10.3%). There were also observable gender differences in mental health profiles, with men more often in the ‘flourishing, no life stress’ group (28.8% vs 22.9%), or in the ‘moderate mental health and stress’ group (28.9% vs 15.5%), whereas women were more often in the ‘flourishing, some life stress’ group (47.3% vs 41.3%) or the ‘clinical mood disorder’ group (7.7% vs 4.5%).

**Table 1:** Sociodemographci characgeristics of the sample by gender.

		Women (N = 1263)	Men (N = 1195)	Overall (N = 2458)
Age (years)	Mean (SD)	45.6 (11.7)	45.9 (11.3)	45.7 (11.5)
	Median (Min, Max)	46.0 (25.0, 64.0)	46.0 (25.0, 64.0)	46.0 (25.0, 64.0)
Age group	25–34	298 (23.6%)	234 (19.6%)	532 (21.6%)
	35–44	277 (21.9%)	326 (27.3%)	603 (24.5%)
	45–54	321 (25.4%)	287 (24.0%)	608 (24.7%)
	55–64	367 (29.1%)	348 (29.1%)	715 (29.1%)
Geographic region	Population centre	1062 (84.1%)	982 (82.2%)	2044 (83.2%)
	Rural	201 (15.9%)	213 (17.8%)	414 (16.8%)
Highest education level	Less than secondary school graduation	71 (5.6%)	74 (6.2%)	145 (5.9%)
	Secondary school graduation	123 (9.7%)	151 (12.6%)	274 (11.1%)
	Some post-secondary	43 (3.4%)	42 (3.5%)	85 (3.5%)
	Post-secondary graduation	973 (77.0%)	867 (72.6%)	1840 (74.9%)
Married/Common-Law		868 (68.7%)	791 (66.2%)	1659 (67.5%)
Employment type	Full-time	722 (57.2%)	889 (74.4%)	1611 (65.5%)
	Part-time	168 (13.3%)	65 (5.4%)	233 (9.5%)
	Presumed unemployed	370 (29.3%)	240 (20.1%)	610 (24.8%)
Income	<$40,000	365 (28.9%)	272 (22.8%)	637 (25.9%)
	$40–59,999	219 (17.3%)	219 (18.3%)	438 (17.8%)
	$60–79,999	198 (15.7%)	182 (15.2%)	380 (15.5%)
	$80–99,999	150 (11.9%)	162 (13.6%)	312 (12.7%)
	$100–149,999	197 (15.6%)	220 (18.4%)	417 (17.0%)
	=>$150,000	134 (10.6%)	140 (11.7%)	274 (11.1%)

Ethnicity (all that apply)	White	986 (78.1%)	932 (78.0%)	1918 (78.0%)
	Black	43 (3.4%)	32 (2.7%)	75 (3.1%)
	East or Southeast Asian	79 (6.3%)	76 (6.4%)	155 (6.3%)
	Latinx	28 (2.2%)	17 (1.4%)	45 (1.8%)
	Middle Eastern	20 (1.6%)	21 (1.8%)	41 (1.7%)
Recent immigrant (<10 years)		90 (7.1%)	68 (5.7%)	158 (6.4%)
Any physical chronic condition	Yes	714 (56.5%)	609 (51.0%)	1323 (53.8%)

**Table 2:** Mental health profiles and mental health service use by gender.

			Women (N = 1263)	Men (N = 1195)	Overall (N = 2458)	Standardized Difference
Employment profile	Job control (employed)	Q1 (Highest)	193 (15.3%)	269 (22.5%)	462 (18.8%)	0.32
		Q2	233 (18.4%)	232 (19.4%)	465 (18.9%)	
		Q3	210 (16.6%)	244 (20.4%)	454 (18.5%)	
		Q4 (lowest)	245 (19.4%)	201 (16.8%)	446 (18.1%)	
	Job search status (unemployed)	Looking for work	61 (4.8%)	58 (4.9%)	119 (4.8%)	
		Not looking for work	246 (19.5%)	123 (10.3%)	369 (15.0%)	
		Unable to work	63 (5.0%)	59 (4.9%)	122 (5.0%)	

Mental health profile cluster		Cluster 1- Flourishing, no life stress	289 (22.9%)	344 (28.8%)	633 (25.8%)	0.2
		Cluster 2 - Flourishing, some life stress	597 (47.3%)	493 (41.3%)	1090 (44.3%)	
		Cluster 3- Moderate mental health and stress	191 (15.1%)	226 (18.9%)	417 (17.0%)	
		Cluster 4- Clinical mood disorder	97 (7.7%)	54 (4.5%)	151 (6.1%)

**Table 3:** – Causal decomposition results, risk difference (RD) per 1000 population of each outcome among women compared to men.

	Natural Course - RD (95% CI)	Counterfactual - RD (95% CI)	% Change (from natural to counterfactual)
Outcome 1- Flourishing, no life stress	−62.8 (−92,5, −32.5)	−67.1 (−97.6, −34.7)	−6.8% (−31.1%, 7.3%)
Outcome 2 - Flourishing, some life stress	66.9 (34.2, 103.4)	86.6 (53.1, 126.5)	−29.4% (−75.7%, −12.1%)
Outcome 3- Moderate mental health and stress	−40.9 (−76.6, −10.1)	−51.4 (−88.2, −24.9)	−25.6% (−144.1%, −8.0%)
Outcome 4- Clinical mood disorder	36.8 (20.4, 53.5)	31.9 (15.6, 52.5)	13.4% (−5.1%, 32.3%)

Among the three groups who were largely absent of a diagnosable mental health disorder, pre-existing gender differences identified above were projected to increase if women had the same employment distribution as men. The ‘flourishing with no life stress group’ contained a lower number of women relative to men (62.8 per 1000 fewer), when women were assigned men's distribution of employment profiles the number of women reduced further by 6.8% (95% CI: reduced by 31.1% to increased by 7.3%). The ‘flourishing with some life stress’ group contained a higher number of women relative to men (66.9 per 1000 higher), when women were assigned men's employment distribution, the number of women relative to men in this group increased further by 29.4% (95% CI: increased by 12.1% to increased by 75.7%). The ‘moderate mental health and stress’ group contained a lower number of women relative to men (40.9 per 1000 fewer), when women were given men's employment distribution, even fewer women were in this group, reduced by 25.6% (95% CI: reduced by 144.1% to reduced by 8.0%) (see Table 3).

Whereas among those who did have a diagnosable mental health disorder, assigning women to have the same employment distribution as men decreased pre-existing gender differences. There were a higher number of women relative to men in the ‘clinical mood disorder’ group (36.8 per 1000 more). When women were assigned men's employment distribution, fewer women were in this group. Gender differences in employment therefore explained 13.4% (95% CI: −5.1%, 32.3%) of the gender differences in the clinical mood disorder profile.

## 4 Discussion

This study advances our understanding of gender differences in mental health in two important ways. First using a multifaceted approach to capturing mental health profiles allowed us to explore the extent to which employment contributes to observed differences in mental health beyond just disorders. Secondly by structuring this as a causal question we can more clearly articulate the influence of gender. We found that among groups who were deemed to be flourishing in their positive mental health, men were more likely to exhibit no life stress, while women were more likely to exhibit at least some life stress. Among groups who exhibited poorer mental health, women were more likely to be among a group meeting the clinical threshold for a mood disorder, while men were more likely to exhibit subthreshold symptoms of poor mental health paired with absence of flourishing.

When women were assigned men's distribution of employment profiles, which in this sample equated to a greater number of women being employed, and employed in jobs with higher quality work, the number of clinical mood disorders among women decreased, reducing the gender disparity in this group. This finding suggests that employment is a contributor to gender differences in mental illness. This is well supported by existing research that has linked unemployment and low levels of job control with poor mental health outcomes including depression (Amiri, 2022; Bonde, 2008).

In contrast, among the three mental health profiles that were largely absent of a diagnosable mental health disorder, when women were assigned the employment distribution of men, pre-existing gender differences increased. Women were more likely to be flourishing with some life stress than men, and even more women entered this group after being assigned the employment profile distribution of men. In contrast women were less likely to have moderate mental health and stress than men and even fewer women were in this group after being assigned the employment profile distribution of men. This suggests that, rather than explaining them, gender differences in employment are suppressing gender differences in well-being, including positive mental health, self-rated mental health, and life stress.

The reasons for this are complex and must be understood with reference to the North American sociocultural context. Research has found that in cultures with increasing gender equality such as North America, women's happiness and life satisfaction has worsened relative to men, this has been referred to as the ‘paradox of declining female happiness’ (Blanchflower & Bryson, 2024; Meisenberg & Woodley, 2015; Stevenson & Wolfers, 2009; Zuckerman, Li, & Diener, 2017). Our findings may be interpreted in the context of this trend, suggesting that as more women enter the labour market and/or inhabit roles with higher levels of control, life stress and well-being may also decrease.

One potential reason for this is that, increasing women in the workforce without eradicating gendered expectations of roles outside of work including caregiving and household responsibilities could exacerbate stress and erode well-being among women. This could also be extended to job control, which potentially comes with increased demands and responsibilities at work that may lie in contrast to gendered roles and expectations both within and outside of work. More recent work has shown that the COVID-19 pandemic has exacerbated the trend of declining happiness for women, in part due to competing demands on women's time in the absence of traditional supports for household and childcare responsibilities (Blanchflower & Bryson, 2024; Giurge, Yemiscigil, Sherlock, & Whillans, 2020). It is important that future work builds upon our findings with more recent data to observe how the relationship between gender, job control and mental health/well-being may have changed over time, given the effects of the COVID-19 pandemic on working lives, particularly considering enduring changes such as remote work.

Strengths of this work include our use of multifaceted measures of both employment and mental health to better capture complexity and gain more accurate estimates of the explanatory effect of employment in gender differences in mental health. We also used a household-based sample of Ontario residents, to represent a diverse range of working adults in this population. Finally, we used a causal decomposition approach to achieve a causal estimate, representing the novel application of this approach within this context.

This study also had some limitations. Firstly, there are important limitations in our ability to capture the continuum of gender. We had access to an interviewer assessed binary gender measure, which does not necessarily represent an individual's current gender identity, or the potentially fluid nature of this concept. In addition, this measure does not capture gender outside of the binary categories of man or woman. The result of this is some misclassification in the exposure variable. In addition, understanding employment experiences among gender minorities and how this relates to mental health likely requires dedicated study with targeted data collection methods that capture diverse gender experiences.

We also had only a single timepoint of data, meaning there is the potential for reverse causality in the relationship between employment and mental health; whereby those with poorer mental health could have an inability to work, or to hold higher quality jobs. It is expected that the highest risk of this is for the group with mental health disorders, who are most likely to be impacted in their ability to obtain and maintain employment. Lacking flourishing or experiencing life stress may be more fluid experiences that are less likely to have long term impacts on employment. Future work with longitudinal measures is required to confirm these findings.

The CCHS-MH does not capture externalizing symptoms and behaviours with the same depth that it captures internalizing behaviours, and these symptoms are more typical of men than women (Kessler & Zhao, 1999; Seedat et al., 2009). Therefore, we may not be able to fully capture mental health problems that may increase when assigning women to men's employment distribution.

Finally, it is important to consider the extent to which this study meets requirements for causality. There are some potential limitations in our ability to talk causally, related to imprecision in the measurement of some confounders such as physical health, and in the consistency assumption, given the aforementioned limitations of the gender measure. This is discussed in detail in Appendix A.

## 5 Conclusion and next steps

To conclude, this study provides a broad estimate of the contribution of employment to gender differences in mental health. Specific next steps include further study of the pathways underpinning gender differences in mental health and well-being using longitudinal data with a broader set of measures to confirm and build upon these findings. Other research may also use this approach with a targeted sampling approach to examine a broader selection of social identities, as well as intersecting social identities. Exploring explanatory factors that may underpin these disparities is informative to different interventions that may seek to reduce them.

## CRediT authorship contribution statement

**Christa Orchard:** Writing – review & editing, Writing – original draft, Visualization, Project administration, Methodology, Funding acquisition, Formal analysis, Data curation, Conceptualization. **Elizabeth Lin:** Writing – review & editing, Supervision, Project administration, Methodology, Data curation, Conceptualization. **Laura Rosella:** Writing – review & editing, Supervision, Methodology, Funding acquisition, Conceptualization. **Peter M. Smith:** Writing – review & editing, Supervision, Methodology, Funding acquisition.

## Ethical statement

Research Ethics Board approval for this study was obtained through the University of Toronto Health Sciences Research Ethics Board (protocol #29510).

## Data availability statement

The dataset from this study is held securely in coded form at ICES. While legal data sharing agreements between ICES and data providers (e.g., healthcare organizations and government) prohibit ICES from making the dataset publicly available, access may be granted to those who meet pre-specified criteria for confidential access, available at www.ices.on.ca/DAS (email: das@ices.on.ca). The full dataset creation plan and underlying analytic code are available from the authors upon request, understanding that the computer programs may rely upon coding templates or macros that are unique to ICES and are therefore either inaccessible or may require modification.

## Funding statement

This study was supported by 10.13039/100012665ICES, which is funded by an annual grant from the Ontario Ministry of Health (MOH) and the Ministry of Long-Term Care (MLTC). This study also received funding from the Data Sciences Institute and the Institute for Work & Health.

## Declaration of competing interest

The authors declare that they have no known competing financial interests or personal relationships that could have appeared to influence the work reported in this paper.

## Data Availability

The data that has been used is confidential.

## References

[bib1] Amick B.C., McDonough P., Chang H., Rogers W.H., Pieper C.F., Duncan G. (2002). Relationship between all-cause mortality and cumulative working life course psychosocial and physical exposures in the United States labor market from 1968 to 1992. Psychosomatic Medicine.

[bib2] Amiri S. (2022). Unemployment associated with major depression disorder and depressive symptoms: A systematic review and meta-analysis. International Journal of Occupational Safety and Ergonomics.

[bib3] Amroussia N., Gustafsson P.E., Mosquera P.A. (2017). Explaining mental health inequalities in northern Sweden: A decomposition analysis. Global Health Action.

[bib4] Batz-Barbarich C., Tay L., Kuykendall L., Cheung H.K. (2018). A meta-analysis of gender differences in subjective well-being: Estimating effect sizes and associations with gender inequality. Psychological Science.

[bib5] Blanchflower D.G., Bryson A. (2024). The female happiness paradox. Journal of Population Economics.

[bib6] Bonde J. (2008). Psychosocial factors at work and risk of depression: A systematic review of the epidemiological evidence. British Medical Journal.

[bib7] Brisson C., Larocque B. (2001). [Validity of occupational stress and decision latitude on health in the National Population Health Survey of 1994-95]. Canadian journal of public health = Revue canadienne de sante publique.

[bib8] Cabezas-Rodríguez A., Utzet M., Bacigalupe A. (2021). Which are the intermediate determinants of gender inequalities in mental health?: A scoping review. International Journal of Social Psychiatry.

[bib9] Drolet M. (2022). Unmasking differences in women's full-time employment. Statistics Canada Catalogue no. 75-006-X.

[bib10] Fleury M.-J., Ngui A.N., Bamvita J.-M., Grenier G., Caron J. (2014). Predictors of healthcare service utilization for mental health reasons. International Journal of Environmental Research and Public Health.

[bib11] Frank J., Mustard C., Smith P., Siddiqi A., Cheng Y., Burdorf A. (2023). Work as a social determinant of health in high-income countries: Past, present, and future. The Lancet.

[bib12] Gedikli C., Miraglia M., Connolly S., Bryan M., Watson D. (2023). The relationship between unemployment and wellbeing: An updated meta-analysis of longitudinal evidence. European Journal of Work & Organizational Psychology.

[bib13] Gilmour H. (2014). Positive mental health and mental illness. Health Reports.

[bib14] Giurge L.M., Yemiscigil A., Sherlock J., Whillans A.V. (2020).

[bib15] Grant F., Guille C., Sen S. (2013). Well-being and the risk of depression under stress. PLoS One.

[bib16] Hwang S., Shin H. (2023). Gender gap in mental health during the COVID-19 pandemic in South Korea: A decomposition analysis. International Journal of Environmental Research and Public Health.

[bib17] Jackson J.W., VanderWeele T.J. (2018). Decomposition analysis to identify intervention targets for reducing disparities. Epidemiology.

[bib18] Kessler R.C., Zhao S., Aneshensel C.S., Phelan J.C. (1999). Handbook of the sociology of mental health.

[bib19] Keyes C.L.M., Eisenberg D., Perry G.S., Dube S.R., Kroenke K., Dhingra S.S. (2012). The relationship of level of positive mental health with current mental disorders in predicting suicidal behavior and academic impairment in college students. Journal of American College Health.

[bib20] Keyes C.L.M., Simoes E.J. (2012). To flourish or not: Positive mental health and all-cause mortality. American Journal of Public Health.

[bib21] Meisenberg G., Woodley M.A. (2015). Gender differences in subjective well-being and their relationships with gender equality. Journal of Happiness Studies.

[bib22] Milner A., Disney G., Byars S., King T.L., Kavanagh A.M., Aitken Z. (2020). The effect of gender on mental health service use: An examination of mediation through material, social and health-related pathways. Social Psychiatry and Psychiatric Epidemiology.

[bib23] Moyser M. (2017).

[bib24] Niedhammer I., Bertrais S., Witt K. (2021). Psychosocial work exposures and health outcomes: A meta-review of 72 literature reviews with meta-analysis. Scandinavian Journal of Work, Environment & Health.

[bib25] OECD (2013).

[bib26] Peckham T., Seixas N., de Castro A.B., Hajat A. (2022). Do different patterns of employment quality contribute to gender health inequities in the U.S.? A cross-sectional mediation analysis. International Journal of Environmental Research and Public Health.

[bib27] Platt J.M., Bates L.M., Jager J., McLaughlin K.A., Keyes K.M. (2020). Changes in the depression gender gap from 1992 to 2014: Cohort effects and mediation by gendered social position. Social Science & Medicine.

[bib28] Provencher H.L., Keyes C.L.M. (2011). Complete mental health recovery: Bridging mental illness with positive mental health. Journal of Public Mental Health.

[bib29] Schomaker M., Heumann C. (2018). Bootstrap inference when using multiple imputation. Statistics in Medicine.

[bib30] Seedat S., Scott K.M., Angermeyer M.C., Berglund P., Bromet E.J., Brugha T.S., Kessler R.C. (2009). Cross-national associations between gender and mental disorders in the world health organization world mental health surveys. Archives of General Psychiatry.

[bib31] Slavich G.M. (2016). Life stress and health: A review of conceptual issues and recent findings. Teaching of Psychology.

[bib32] Statistics Canada (2012). Canadian community health survey, 2012: Ment health. https://www23.statcan.gc.ca/imdb/p2SV.pl?Function=getSurvey&Id=119789.

[bib33] Stevenson B., Wolfers J. (2009). The paradox of declining female happiness. American Economic Journal: Economic Policy.

[bib34] Stier H., Yaish M. (2014). Occupational segregation and gender inequality in job quality: A multi-level approach. Work, Employment & Society.

[bib35] Sudharsanan N., Bijlsma M.J. (2021). Educational note: Causal decomposition of population health differences using Monte Carlo integration and the g-formula. International Journal of Epidemiology.

[bib36] Van der Doef M., Maes S. (1999). The job demand-control (-Support) model and psychological well-being: A review of 20 years of empirical research. Work & Stress.

[bib37] VanderWeele T.J., Robinson W.R. (2014). On the causal interpretation of race in regressions adjusting for confounding and mediating variables. Epidemiology.

[bib38] Zuckerman M., Li C., Diener E.F. (2017). Societal conditions and the gender difference in well-being: Testing a three-stage model. Personality and Social Psychology Bulletin.

