# Supplementary material for: Using a causal decomposition approach to estimate the contribution of employment to differences in mental health profiles between men and women
Source: SSM Popul Health. 2024 Oct 12;28:101718. doi: 10.1016/j.ssmph.2024.101718 (PMC11615145; doi:10.1016/j.ssmph.2024.101718)
Supplement: Multimedia component 1 [file mmc1.docx]

Appendix A - Causal assumptions

1. Conditional exchangeability

The assumption of conditional exchangeability for the group and explanatory variable refers in this case to the fact that, given confounders of the gender-mental health and employment-mental health pathways, assignment of gender is independent of the potential outcomes. In other words, if we have accounted for all of the confounders of these relationships, then the only effect of gender on mental health profiles is due to gender itself. In our case, there can be no true confounders of the gender-mental health relationship given that no factor can cause a person’s gender identity. Therefore most variables are downstream of gender and upstream of mental health. However, within this sample, it may be the case that there is a relationship between gender and other sociodemographic characteristics such as ethnicity and age due to differential likelihood of participation among these groups, for example more older women may participate compared to older men, creating a relationship between age and gender among the included sample. This is not expected to be large given the sampling design of the CCHS-MH, however since we did not use sampling weights we chose to account for this.

In contrast there are potential factors that could confound the relationship between employment and mental health, including sociodemographic characteristics such as age, ethnicity and geographic region, as well as factors that influence both likelihood of attaining high quality employment that also influence mental health including physical health and educational attainment. We were able to account for these confounders as they were captured in the CCHS. However, we may not capture them at a high level of specificity due to the sample size, for example we did not have enough sample to adequately represent all ethnicity groups, and we could not differentiate across different types of physical conditions to capture things like disability and functioning.

1. Positivity

Positivity refers to the assumption that all individuals across all levels of confounding covariates have the chance to experience all levels of the grouping variable and the explanatory variable. In our case, all groups within the population can be a man or a woman, and we do not expect that there are any groups who are entirely unable to work or work in higher quality jobs. While it may be possible that there are individuals with severe and debilitating chronic conditions, none of the chronic conditions we captured would entirely prevent an individual from working, and it is unlikely that we captured any individuals who were completely unable to work in the CCHS-MH sample given these individuals are less likely to be household dwelling and/or able to participate in a survey interview.

1. Consistency

Consistency refers to the assumption that there is a clear and well-defined treatment, or exposure, that results in a consistent effect of the grouping variable and the explanatory variable on the outcome. In our case, being a man compared to a woman is clearly defined, there is variation among these groups due to capturing a broader concept of gender into a simplified binary categorisation. In addition, our employment measure is multidimensional and captures employment in a broader way than most studies that simply look at it as a binary variable, however we do not directly capture other aspects of employment that could be linked to mental health such as income.

Appendix B – Measurement of employment profiles

Description of employment measure selection and alternative candidates considered:

Job control can cut across different all employment experiences, as well as being a central component of the individual’s nature of work. Other important dimensions of work which are captured in the 2012 CCHS-MH survey also include personal income, part- vs full-time work, or some grouping of occupational hazards using an individual’s occupational classification. Income is an aspect of employment relating to material resources which is linked to mental health, however most of the gender disparity is in personal income, whereas material resources and potential deprivation are best captured by household income (Pelletier & Patterson, 2019; Public Health Agency of Canada, 2022) . In addition, while personal income could act as a proxy for job quality, this would also be imperfect due to differences in pay across economic sectors and occupations, therefore job control is a more direct measure of this. There are gender differences in occupation that could predispose men or women to certain psychosocial exposures, for example women more often work in the healthcare industry and therefore may be exposed to harassment and workplace violence (Shea, Sheehan, Donohue, Cooper, & De Cieri, 2017; Statistics Canada, 2023). However, there are important variations within occupational groups in quality of work, as well as differences between men and women in terms of exposure to hazards within occupations that would not be captured using this classification (Biswas et al., 2021). Finally, women are more likely to seek jobs that offer more flexibility and work-life balance, including part-time jobs, and these jobs are also often of lower quality (Stier & Yaish, 2014). We believe it is likely that lower quality jobs will be captured as those exhibiting ‘low job control’. However, other aspects of part-time work such as job security and access to benefits, while likely correlated with job control require dedicated study.

Appendix table B.1 – Employment profiles

The following table includes the CCHS-MH items used to create the employment profile:

| Variable name | Concept | Question | Response options |
| --- | --- | --- | --- |
| lbs_01 | Worked at job or business last week | Last week, did you work at a job or a business? Please include part-time jobs, seasonal work, contract work,  self-employment, baby-sitting and any other paid work, regardless of the number of hours worked | Yes/No/Permanently unable to work |
| lbs_02 | Absent from job or business last week | Last week, did you have a job or business from which you were absent? | Yes/No |
| lbs_11 | Looked for work in past 4 weeks | In the past 4 weeks, did you do anything to find work? | Yes/No |
| WST_401 | Work stress - learn new things | The next few questions are about your main job or business in the past 12 months. I’m going to read you a  series of statements that might describe your job situation. ... Your job required that you learn new things. | Rating 1 (strongly agree) to 5 (strongly disagree) |
| WST_402 | Work stress - high level of skill | The next few questions are about your main job or business in the past 12 months. I’m going to read you a  series of statements that might describe your job situation. ... Your job required a high level of skill. | Rating 1 (strongly agree) to 5 (strongly disagree) |
| WST_403 | Work stress - freedom to decide | The next few questions are about your main job or business in the past 12 months. I’m going to read you a  series of statements that might describe your job situation. ... Your job allowed you freedom to decide how  you did your job. | Rating 1 (strongly agree) to 5 (strongly disagree) |
| WST_404 | Work stress - repetitive tasks | The next few questions are about your main job or business in the past 12 months. I’m going to read you a  series of statements that might describe your job situation. ... Your job required that you do things over  and over. | Rating 1 (strongly agree) to 5 (strongly disagree) |
| WST_409 | Work stress - had own job input | The next few questions are about your main job or business in the past 12 months. I’m going to read you a  series of statements that might describe your job situation. ... You had a lot to say about what happened  in your job. | Rating 1 (strongly agree) to 5 (strongly disagree) |

Appendix table B.2.

This table indicates how the items in the previous tables were used to create the 7 employment profile levels used in this analysis:

| Level | Requirements |
| --- | --- |
| Unemployed, not seeking work | If lbs_01 = ‘no’ and lbs_02 = ‘no’ and lbs_11 = ‘no’ |
| Unemployed, seeking work | If lbs_01 = ‘no’ and lbs_02 = ‘no’ and lbs_11 = ‘yes’ |
| Unemployed, unable to work | If lbs_01 = ‘permanently unable to work’ |
| Employed, job control quartile 1 | If lbs_01 = ‘yes’ or lbs_02 = ‘yes’  and  summed wst_401, wst_402, wst_403, wst_404 (reverse coded), wst_409, then split into quartiles |
| Employed, job control quartile 2 |  |
| Employed, job control quartile 3 |  |
| Employed, job control quartile 4 |  |

Appendix C – Measurement of mental health profiles

Appendix table C.1. Mental health profiles

This table contains the measures in the CCHS-MH used to create the mental health profiles.

| Concept | Instrument (if applicable) | Description |
| --- | --- | --- |
| Mood disorder | World Mental Health-Composite International Diagnostic Interview (WMH-CIDI) instrument | Met Diagnostic Statistical Manual-IV criteria for past-year depression and/or bipolar disorder |
| Anxiety disorder | WMH-CIDI instrument | Met DSM-IV criteria for past-year generalized anxiety disorder |
| Substance use disorder | WMH-CIDI instrument | Met DSM-IV criteria for past-year alcohol, cannabis or other substance abuse and dependence |
| Schizophrenia or psychosis |  | Single-item self-reported current diagnosis |
| Eating disorder |  | Single-item self-reported current diagnosis |
| Suicidal thoughts or attempts in past 12 months |  | Single items indicating serious thoughts, attempts or plans for suicide in the past 2 weeks or past 12 months |
| Psychological distress | Kessler 6-item psychological distress scale |  |
| Self-rated mental health |  | Single-item general self-assessment of mental health |
| Life stress |  | Single item amount of stress in life on most days |
| Positive mental health | Mental Health Continuum short-form (MHC-SF) | Continuous score used to classify individuals in one of 3 groups |

Analysis description: In order to identify clusters with similar mental health profiles, a Partitioning Around Medoids (PAM) algorithm was used, a centroid-based clustering algorithm that uses an iterative process to identify clusters. The Gower’s proximity function was used to define proximity between each individual and the cluster centre, as it adapts to different data types including continuous, ordinal and nominal/binary data, to produce an overall measure of distance between 0 (identical) and 1 (maximally dissimilar). This algorithm was repeated with between 2 and 8 clusters specified, and the cluster solution with the best performance (using quantitative indicators of clinical validity as well as assessment of the utility of the solutions), was selected. The four cluster solution selected is displayed in the main body of the manuscript.

This analytical work is described in greater detail elsewhere (Orchard, Lin, Rosella, & Smith, 2024)

Appendix References;

Biswas, A., Harbin, S., Irvin, E., Johnston, H., Begum, M., Tiong, M., . . . Smith, P. (2021). Sex and Gender Differences in Occupational Hazard Exposures: a Scoping Review of the Recent Literature. *Current Environmental Health Reports, 8*(4), 267-280. doi:10.1007/s40572-021-00330-8

Orchard, C., Lin, E., Rosella, L., & Smith, P. M. (2024). Using unsupervised clustering approaches to identify common mental health profiles and associated mental health-care service-use patterns in Ontario, Canada. *American Journal of Epidemiology, 193*(7), 976-986. doi:10.1093/aje/kwae030

Pelletier, R., & Patterson, M**.** (2019). *The gender wage gap in Canada: 1998 to 2018*. Statistics Candada

Public Health Agency of Canada. (2022). *Mental health inequalities by income in Canada*. Retrieved from <https://www.canada.ca/en/public-health/services/publications/science-research-data/mental-health-inequalities-income-canada.html>

Shea, T., Sheehan, C., Donohue, R., Cooper, B., & De Cieri, H. (2017). Occupational Violence and Aggression Experienced by Nursing and Caring Professionals. *Journal of Nursing Scholarship, 49*(2), 236-243. doi:<https://doi.org/10.1111/jnu.12272>

Statistics Canada. (2023). Table: 14-10-0335-02 Proportion of women and men employed in occupations, annual, inactive.

Stier, H., & Yaish, M. (2014). Occupational segregation and gender inequality in job quality: a multi-level approach. *Work, Employment and Society, 28*(2), 225-246. doi:10.1177/0950017013510758
